# Anatomical variants of the posterior horns of the lateral ventricles: an MRI study

**DOI:** 10.3389/fnimg.2025.1478137

**Published:** 2025-04-07

**Authors:** Ronen Spierer, Omer Zarrabi Itzhak, Jonathan Gross, Tamer Sobeh, Shai Shrot

**Affiliations:** ^1^Rappaport Faculty of Medicine, Technion-Israel Institute of Technology, Haifa, Israel; ^2^Faculty of Medical and Health Sciences, Tel Aviv University, Tel Aviv, Israel; ^3^Department of Diagnostic Imaging, Sheba Medical Center, Ramat-Gan, Israel

**Keywords:** neuroanatomy, anatomical variations, MRI, lateral ventricles, posterior horn

## Abstract

**Introduction:**

Anatomical variations in the posterior horns of the lateral ventricles are well-documented, with the horn presenting as open, constricted, or completely closed. However, the extent and nature of these variations across different demographics remain under-explored. This study aimed to investigate the anatomical variations of the posterior horn of the lateral ventricles across different age and sex groups and to compare the variations between the right and left sides.

**Methods:**

We conducted a retrospective analysis of magnetic resonance imaging (MRI) scans from 217 adult participants across 15 age groups, utilizing a stratified random sampling from a radiology database. MRI scans were analyzed for ventricular dimensions, and horn types (open, constricted, and closed). Statistical significance was defined as *p*-value < 0.05.

**Results:**

Variants of the posterior horn were observed frequently, with open posterior horn being the most common in the left lateral ventricle (41%) and constricted type being the most common in the right lateral ventricle (37%). A significant correlation existed between the right and left horn types, but in most cases, there was a difference in type between the right and the left horns in the same individual. No significant association between age and the type of the posterior horns was found. However, there was a significant difference in the width and length of the horns between the open and other types, with open horns being wider and longer. Lastly, the left horn appeared longer than the right one.

**Discussion:**

The findings underline the high variability in posterior horn morphology, which is not significantly influenced by age or sex but varies between individuals and sides. Future studies should explore the functional impact of these anatomical variations.

## Introduction

The ventricular system of the brain comprises four interconnected cavities known as ventricles, filled with cerebrospinal fluid (CSF). The system includes the two lateral ventricles, the midline-located third ventricle, and the fourth ventricle, which is positioned at the level of the brainstem. CSF is produced mainly by the choroid plexus in the ventricles and eventually escapes the system from the three apertures of the fourth ventricle into the subarachnoid space, where it can be absorbed (Shenoy and Lui, 2022; Spierer, 2023; Yamada and Mase, 2023).

The lateral ventricles, being the largest, produce most of the CSF (Adigun and Al-Dhahir, 2024). Ventricular enlargement occurs due to normal aging but is also associated with many pathological conditions, such as hydrocephalus, Alzheimer's disease, and schizophrenia. The volumes of the lateral ventricles are often used as a marker for cerebral atrophy (LeMay, 1984; Kaye et al., 1992; Wilms et al., 1992).

Anatomically, each lateral ventricle comprises a body, an atrium, and three horns: frontal, inferior (temporal), and posterior (occipital). The lateral ventricles may exhibit asymmetry, which, according to a few studies, can be influenced by handedness or sex. However, the clinical significance of these asymmetries remains a matter of debate. A recent review by Scelsi et al. (2020) claimed that the link between ventricular asymmetry and diseases is still not fully understood.

According to Gray's Anatomy, the posterior horn curves medially and impinges deep into the occipital lobe of the cerebrum. It is bounded by a roof and a lateral wall, formed by the tapetum of the corpus callosum, which separates the ventricle from the optic radiation and the inferior fronto-occipital fasciculus, and by a medial wall which is formed by the forceps major superiorly and the calcar avis inferiorly (Standring, 2021).

**Figure 1:**
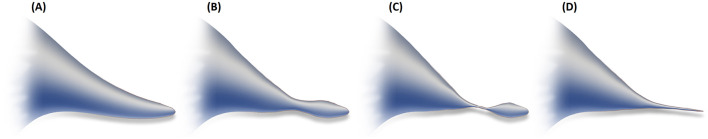
Illustration of a sagittal view of the posterior horn as in anatomy atlases and textbooks **(A)** vs. its actual appearances **(B**–**D)**.

In anatomy textbooks and atlases, the posterior horn is usually drawn as a continuous hollow cone that narrows in diameter as it extends posteriorly to a complete ending. However, this is not the case in actual brains, as there is a significant narrowing at the level of the calcarine sulcus. In addition, in some cases, the horn is completely closed posteriorly to the calcarine sulcus (Curran, 1909). In Gray's Anatomy, while the horn is drawn in only one way, as open, the text notes that it might be absent (Figure 1).

The variations of the posterior horn were first observed by Dalton in 1885 (Dalton, 1885). In 1909, Curran studied these variations in 64 cadavers. He reported that in 24 cases, the horn was open. In 22 cases there was an adhesion of the ependyma, causing a total constriction at the level of the calcarine sulcus but posteriorly to it, the ventricle was patent. The cutting off of the communication between the posterior part and the rest of the ventricle led to an anterior-posterior tandem appearance of the horn. In the remaining 18 cadavers, the horn was completely closed (Curran, 1909). In 1934, another study confirmed the existence of these three variants (Torkildsen, 1934). In these two studies, there was no mention of laterality. Moreover, no comparison between sexes and different ages was made.

**Figure 2:**
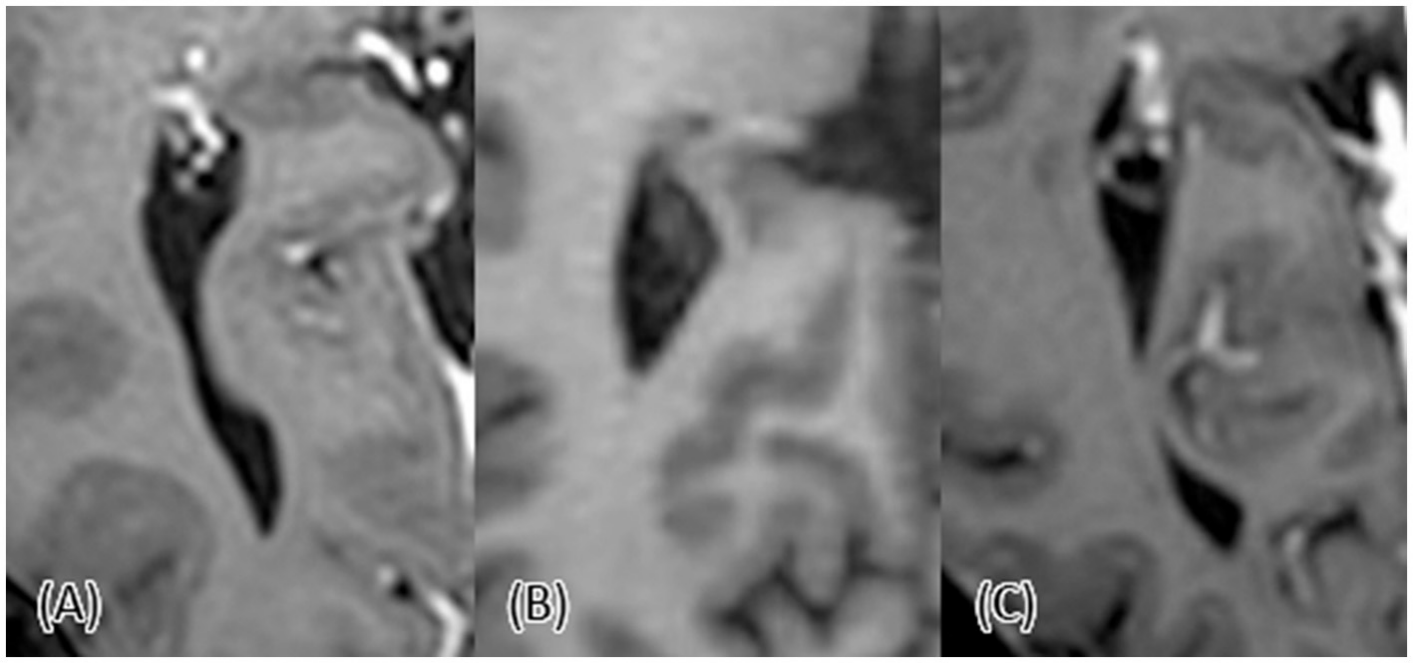
T1-weighted axial view of the posterior horn. **(A)** The posterior horn is open. **(B)** The posterior horn is closed. **(C)** The posterior horn is constricted with a tandem appearance.

These three variants of the posterior horn (open, constricted, and closed) can also be easily seen using modern radiographic tools (Figure 2). Surgically and pathologically, the posterior horn bears clinical significance. It may serve as an anatomical landmark for the optic radiation and allows a surgical route to the ventricle in cases of interventricular hemorrhage (Ebeling and Reulen, 1988; Onoda et al., 2001). Additionally, its enlargement is an important finding in posterior cortical atrophy and related disorders (Nagaratnam and Nagaratnam, 2016). This finding can also be used in differentiating between Alzheimer's disease and dementia with Lewy bodies (Ye et al., 2016). Another noteworthy significance of the posterior horn is in periventricular lesions. It has been previously suggested that such lesions in the posterior horn are specific to multiple sclerosis, in contrast to lesions in other periventricular territories (Casini et al., 2013).

The purpose of the current study was to examine the posterior horn for its anatomical variations in different age groups and to compare between the right and left ventricles.

## Methods

### Study population

This study utilized a retrospective stratified random sampling design across a diverse age spectrum, based on Sheba Medical Center's radiology database. Participants were pooled from 15 distinct age groups, each spanning a five-year bracket, ranging from 20–24 years to 90–94 years. Each age group, except for the 90–94 bracket, included 15 individuals who met the inclusion criteria. Due to the specific challenges associated with recruiting older populations that satisfied our inclusion criteria, the 90–94-year-old age group comprised only 7 participants. We included patients whose clinical reports, as assessed by a specialized neuroradiologist, were normal, with no evidence of neoplastic or neurologic disease history.

### MRI characteristics and analysis

Magnetic resonance imaging (MRI) studies were performed using 3.0 Tesla systems, Ingenia, Philips Medical Systems, Netherlands, or Prisma, Siemens Healthcare, Germany. All patients had routine clinical MRI scans, including T1 and T2 weighted imaging, diffusion-weighted imaging (axial 2D spin-echo sequence with EPI readout, with two b values of 0 and 1,000 mm^2^/s), and susceptibility-weighted imaging.

**Figure 3:**
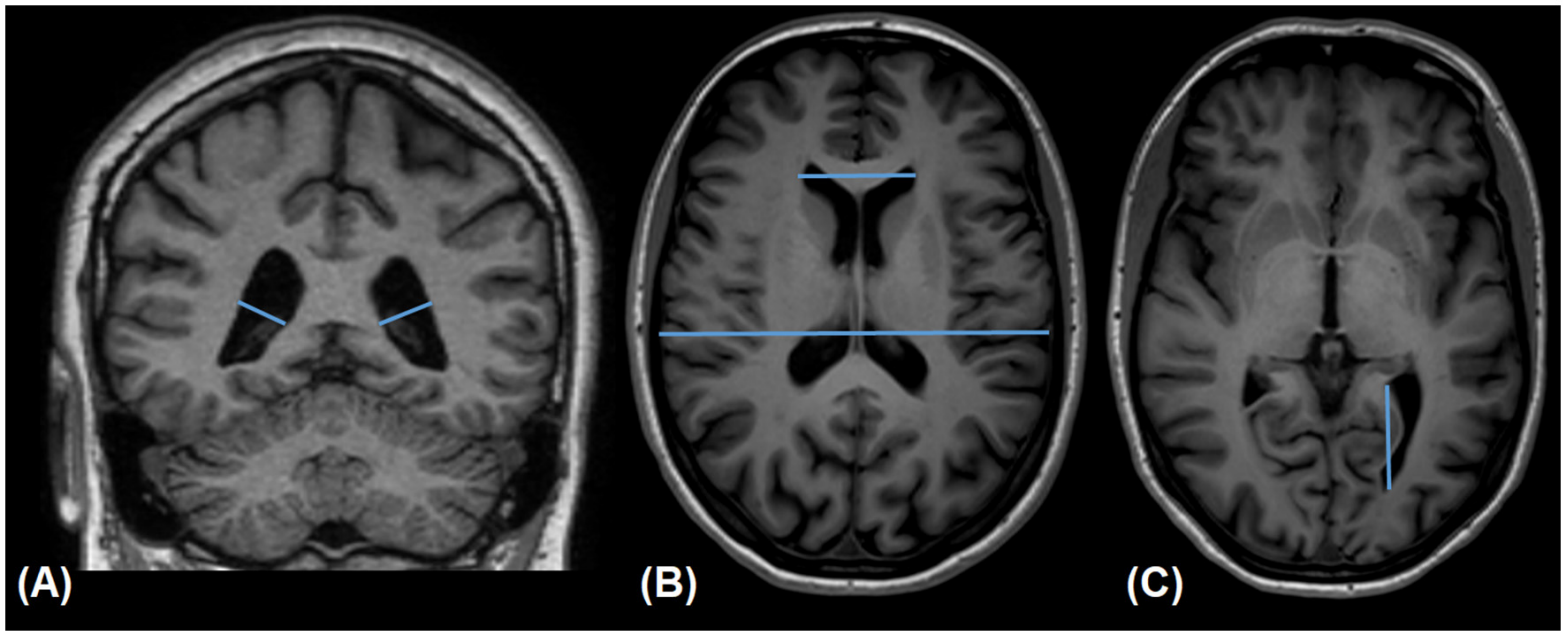
Measurements of the ventricles' width **(A)**, Evans index **(B)**, and the posterior horn's length **(C)**.

Analysis was performed by a single observer on 1 mm volumetric T1 weighted images. The ventricles' type was classified based on an axial view. In cases of doubt, the observer shifted to a coronal view, and a constriction was decided if the ventricle was not seen in at least two consecutive slides. The horn's length was measured as the distance from the atrium to the horn's tip. The ventricle's width was measured at the level of the atrium on coronal reformat as the distance in a superior-lateral axis. For constricted horns, the constriction's length was quantified as the number of consecutive slides, in coronal view, in which the ventricle could not have been seen. The Evans index was measured as the ratio between the maximal distance between the frontal horns and the maximal width of the inner diameter of the skull. Examples of the MRI measurements are shown in Figure 3.

### Statistical analysis

Statistical analyses were performed using SPSS software (IBM, Chicago, USA). All tests performed were two-tailed, and statistical significance was defined as *p*-value < 0.05. All variables were continuous, except for sex and horn type which were categorial, and age group which was ordinal. Categorical variables were compared using the χ^2^ test. A linear-by-linear association χ^2^ test was used to identify trends for different age groups. Correlation was tested using the Pearson correlation test.

For continuous variables, we initially used the D'Agostino-Pearson test for normality, which was positive for the ventricular width. Normally distributed continuous variables are presented as means and standard deviations, and non-normally distributed continuous variables are presented as medians and interquartile ranges (IQRs).

The Mann-Whitney *U* test and the Kruskal-Wallis *H*-test were used for independent samples of widths and the Wilcoxon signed-rank test was used for paired samples (right vs. left ventricles' widths in the same person). The Jonckheere-Terpstra trend test was used to identify age-related trends for width.

Lengths were compared using a paired-samples *t*-test an independent-samples *t*-test was used to compare the Evans index of the open and the other forms of the posterior horn. One-way ANOVA was used to compare the means of the horn's length between different types. For correlating age and horn length, an ANCOVA was used, with horn type as a fixed factor. A linear mixed-effects model was used to compare the length of the right and left horns, controlling for horn type, with side and horn type as fixed effects and to account for intra-participant correlation.

## Results

**Figure 4:**
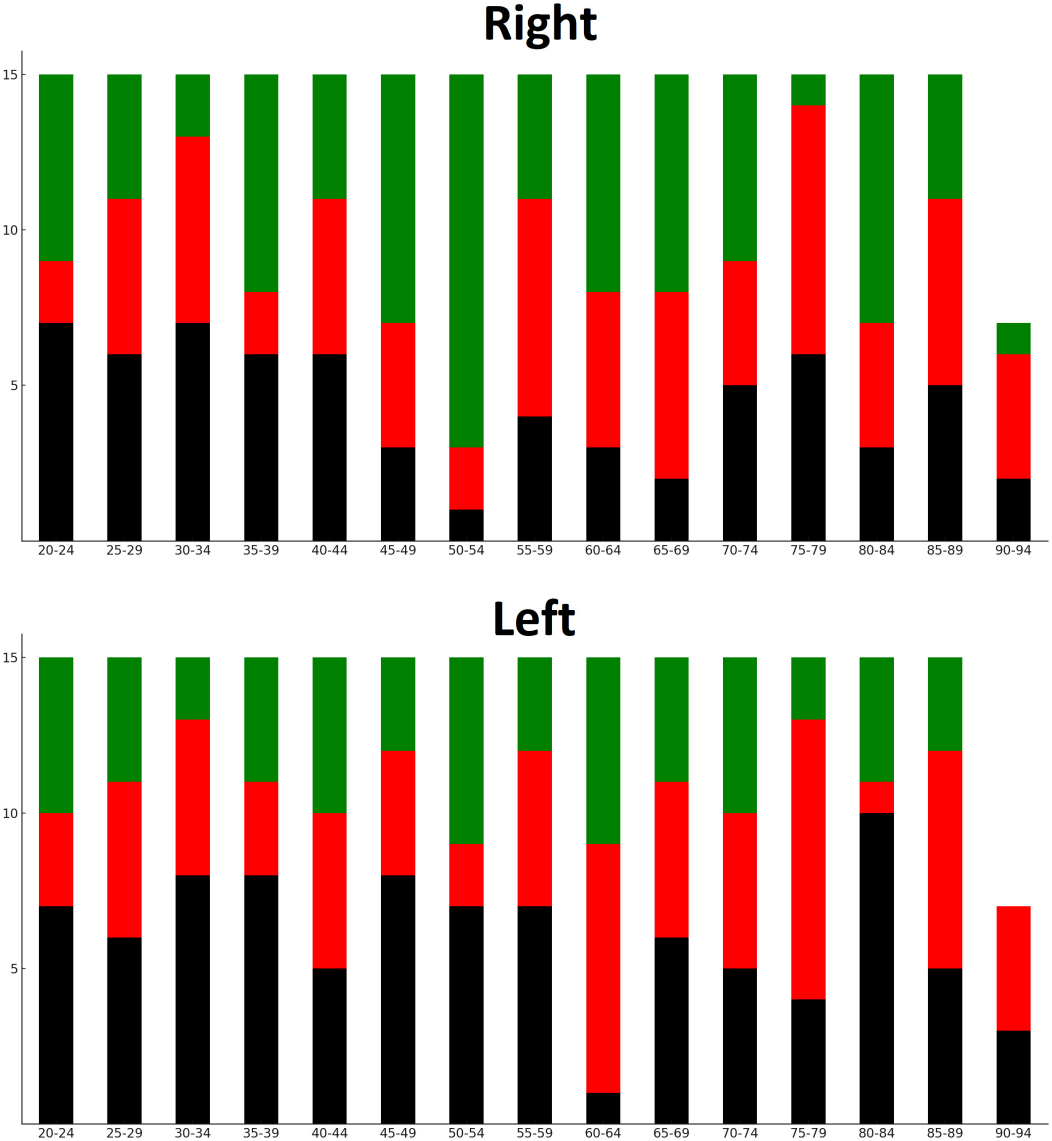
Distribution of horn types among age groups. Black = open; red = closed; green = constricted.

One-hundred-and-two scans of male brains and 115 scans of female brains were included in this study. The distribution of posterior horn types is presented in Table 1. The left posterior horns tended to be open at higher rates than the right ones (*p* = 0.016). A significant correlation was found between the type of the right and left posterior horns (*p* < 0.001). As presented in Figure 4, an open horn type was not associated with age (*p* = 0.098 for the right horn and *p* = 0.379 for the left horn).

**Table 1:** Distribution of the posterior horn types among the study population.

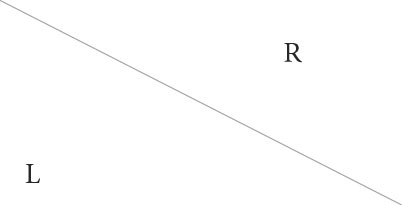	Open 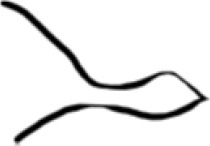	Constricted 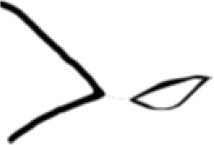	Closed 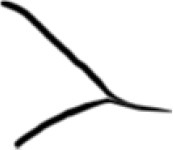	Total
Open 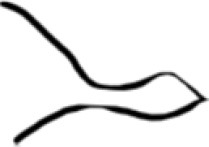	46	32	12	**90**
Constricted 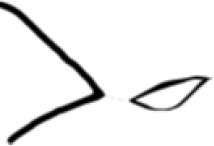	10	36	10	**56**
Closed 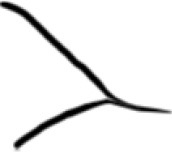	10	13	48	**71**
**Total**	**66**	**81**	**70**	**217**

R = right; L = left.

The median width of both the right and left ventricles was 10 mm (IQR 8–12 mm for both right and left horns; *p* = 0.575). Both ventricular widths were associated with older age (*p* < 0.001), but sex was not associated with a different width (*p* = 0.128 for right; *p* = 0.091 for left).

For the right ventricle, the median width was 11 mm (IQR 9–13mm) in the group of open and 10 mm (IQR 8–11 mm) among the other forms (*p* < 0.001). For the left ventricle, the median width was 11.5 mm (IQR 9–13 mm) in the group of open and 9 mm (IQR 8–11 mm) among the other forms (*p* < 0.001). For both ventricles, no statistically significant difference in width was observed between closed and constricted horns (*p* = 0.355 for right; *p* = 0.503 for left). The mean Evans index did not significantly differ between the open group and the other forms (*p* = 0.066 for the right posterior horn; *p* = 0.207 for the left posterior horn).

The average length of the right ventricle's horn was 33.08 ± 4.47 mm for open, 14.29 ± 3.23 mm for closed, and 32.78 ± 4.14 mm for constricted (*p* < 0.001). The average length of the left ventricle's horn was 33.72 ± 4.99 mm for open, 15.58 ± 3.93 mm for closed, and 33.70 ± 3.66 mm for constricted (*p* < 0.001). The left horn was significantly longer than the right one (*p* < 0.001), a result that remained significant when controlling for horn type (*p* < 0.001). No correlation was found between age and length when controlling for horn type (*p* = 0.365 for right and *p* = 0.696 for left ventricles).

In cases of constriction, the mean constriction was 10.58 ± 4.19 coronal slides for the 81 right horns and 10.98 ± 4.16 slides for the 56 left horns. In the 36 cases where both horns were constricted, the length of constriction appeared correlated (*R* = 0.370; *p* = 0.026), with no significant difference in the lengths of constriction (*p* = 0.761). All statistically significant results are summarized in Table 2.

**Table 2:** Summary of statistically significant findings.

**Measurement**	**Finding**	***p*-value**
Posterior horn type (left vs. right)	Higher rate of open type in the left horn	0.016^a^
Correlation between right and left horn types		< 0.001^a^
Width association with older age (left)		< 0.001^b^
Width association with older age (right)		< 0.001^b^
Ventricular width by horn type (left)	Wider in open horns	< 0.001^c^
Ventricular width by horn type (right)	Wider in open horns	< 0.001^c^
Length by horn type (left)	Closed horns shorter	< 0.001^d^
Length by horn type (right)	Closed horns shorter	< 0.001^d^
Horn's length (left vs. right)	Left longer than right	< 0.001^e, f^
Correlation between right and left lengths of constriction		0.026^g^

^a^χ2 test; ^b^Kruskal-Wallis H test; ^c^Mann-Whitney U test; ^d^One-way ANOVA; ^e^Paired-samples t-test; ^f^ANCOVA; ^g^Pearson's correlation.

## Discussion

This study analyzed 217 brain MRI scans to explore the morphological differences in the posterior horns of the lateral ventricles and their association with age, sex, and bilateral symmetry. The analysis revealed a preference for open types in the left posterior horns compared to the right, alongside a robust correlation between the types on either side. Although no significant sex differences were observed in ventricular width, age was positively associated with increased widths across both sides, as expected. Notably, open posterior horns exhibited greater widths and lengths compared to other forms, suggesting that the degree of openness may significantly influence ventricular dimensions.

The finding that the lateral ventricles' width does not differ between the right and left sides aligns with previous studies that have found no significant difference in their width. Nonetheless, this subject is still debated, and some studies have found that either the right or left ventricle is physiologically larger (Scelsi et al., 2020). The same applies to differences between sexes, which were not found in this study; however, some did describe that men have larger ventricles (Chung et al., 2006).

As expected, ventricular width was associated with older age and with the open type. However, an open type was not more prevalent among the older population. These observations lead us to hypothesize that the ependymal adhesion at the level of the calcarine sulcus may serve as a protective factor against age-related ventricular enlargement in this part of the ventricle. An alternative explanation arises from the idea that age-related ventricular enlargement primarily affects anterior regions, with the occipital lobe being less prone to such changes (Resnick et al., 2003). Either way, given that the Evans index was not associated with one of the types of horn, our findings suggest that while the ventricle may increase in overall size, the type remains unchanged throughout life, even as the ventricle enlarges. The posterior horn was detected longer in the left ventricle and no correlation was found between the length and age. The latter finding is in contrast with a recent study which found a positive correlation between age and length (Rathod and Shembekar, 2018). However, like in our study, they observed the right-left asymmetry, where the left horn is longer. The difference in length was about 1 mm in our study, compared to 2 mm in theirs. A limitation of their study was the use of computed tomography, in which the ventricles can less clearly be seen due to poorer resolution. This may explain both the larger difference in horn length between the ventricles and the correlation with age. As ventricles tend to widen with age, the posterior horn might have appeared longer in elderly subjects due to increased visibility rather than actual elongation. In contrast, we used MRI, which offers higher resolution and allows for clearer visualization of the posterior horn, with and without ventricular dilation. This right-left asymmetry was also observed in a pneumoencephalography study from 1968 (McRae et al., 1968).

A key finding of this study is the asymmetry of the lateral ventricles. While width did not vary between the right and left ventricles, asymmetry was observed in both the length and the structure of the posterior horn. Interestingly, while there was a tendency toward an identical type in each of the two horns, discrepancies were observed in most cases. Moreover, the posterior horn of the left ventricle tended to be open at higher rates than the horn of the right one.

As to the functional impact of the different variants, the literature is sparse. Over a century ago, Curran (1909) proposed a potential link between posterior horn variations and conditions such as seizures and migraines, possibly due to its proximity to the optic radiation. Given the possible involvement of the optic radiation in migraines (Rocca et al., 2008), this is a potential direction for future research. The anatomical variants observed in our study may also have implications in clinical conditions or procedures involving the posterior horn. Whether ependymal adhesions could be disrupted by conditions like hydrocephalus is also a direction for further investigation.

This study has several limitations. An obvious one is the retrospective nature of the study. Also, we included patients from one medical center who underwent an MRI scan due to medical inquiry, and therefore, a selection bias is possible. This could limit the generalizability of our findings, as the patient population may not fully represent broader demographic or clinical characteristics seen in other regions or healthcare settings. Regarding technical limitations, it should be noted that some of the subtle differences between the left and right ventricles approached the resolution limit of the imaging modality (1 mm), which could introduce minor measurement variability and affect the precision of statistical analysis. Additionally, the use of two different MRI instruments within our clinical setting may have further affected measurement consistency and precision. Lastly, the scans were analyzed by one evaluator. In addition to the limitations, since we utilized a stratified sampling design, the data presented here may not reflect the true distribution of the horns within the general population.

In conclusion, this preliminary anatomical study demonstrates substantial variability in the length and shape of the posterior horn of the lateral ventricle, both between individuals and within the same individual. These findings provide foundational data, emphasizing the need for further research to clarify potential clinical implications, particularly regarding neuroimaging interpretation, laterality, CSF dynamics, and neurosurgical planning.

## Data Availability

The raw data supporting the conclusions of this article will be made available by the authors, without undue reservation.
